# Secondary findings in inherited heart conditions: a genotype-first feasibility study to assess phenotype, behavioural and psychosocial outcomes

**DOI:** 10.1038/s41431-020-0694-9

**Published:** 2020-07-20

**Authors:** Elizabeth Ormondroyd, Andrew R. Harper, Kate L. Thomson, Michael P. Mackley, Jennifer Martin, Christopher J. Penkett, Silvia Salatino, Hannah Stark, Jonathan Stephens, Hugh Watkins

**Affiliations:** 1 Division of Cardiovascular Medicine, Radcliffe Department of Medicine, University of Oxford, Oxford, UK; 2 National Institute for Health Research (NIHR) Comprehensive Biomedical Research Centre, Oxford, UK; 3 Wellcome Centre for Human Genetics, University of Oxford, Oxford, UK; 4 Oxford University Hospitals NHS Foundation Trust, Oxford, UK; 5 NIHR BioResource, Cambridge University Hospitals, Cambridge Biomedical Campus, Cambridge, UK

**Keywords:** Genetic testing, Health care

## Abstract

Disclosing secondary findings (SF) from genome sequencing (GS) can alert carriers to disease risk. However, evidence around variant-disease association and consequences of disclosure for individuals and healthcare services is limited. We report on the feasibility of an approach to identification of SF in inherited cardiac conditions (ICC) genes in participants in a rare disease GS study, followed by targeted clinical evaluation. Qualitative methods were used to explore behavioural and psychosocial consequences of disclosure. ICC genes were analysed in genome sequence data from 7203 research participants; a two-stage approach was used to recruit genotype-blind variant carriers and matched controls. Cardiac-focused medical and family history collection and genetic counselling were followed by standard clinical tests, blinded to genotype. Pathogenic ICC variants were identified in 0.61% of individuals; 20 were eligible for the present study. Four variant carriers and seven non-carrier controls participated. One variant carrier had a family history of ICC and was clinically affected; a second was clinically unaffected and had no relevant family history. One variant, in two unrelated participants, was subsequently reclassified as being of uncertain significance. Analysis of qualitative data highlights participant satisfaction with approach, willingness to follow clinical recommendations, but variable outcomes of relatives’ engagement with healthcare services. In conclusion, when offered access to SF, many people choose not to pursue them. For others, disclosure of ICC SF in a specialist setting is valued and of likely clinical utility, and can be expected to identify individuals with, and without a phenotype.

## Introduction

Genome sequencing (GS) has been widely adopted by healthcare systems to investigate genomic contributions to rare disease and cancer [1]. GS coincidentally creates large amounts of data of potential health significance (secondary findings, SF), that are or may be unrelated to the presenting condition. SF are perceived both as an opportunity to predict and prevent disease [2] and a challenge to implementation of genomic medicine [3].

Inherited cardiac conditions (ICC) have a population prevalence of up to one in 200; a proportion result from a single variant in any one of a number of genes. ICC—which include hypertrophic cardiomyopathy (HCM), dilated cardiomyopathy (DCM), arrhythmogenic right ventricular cardiomyopathy (ARVC), long QT syndrome and Brugada syndrome—are often asymptomatic, yet pose a risk of complications including sudden cardiac death. Disease manifestation and risk of complications, including arrhythmogenic events, can be assessed using standard cardiological tests based on imaging and ECG. Evidence-based risk strategies to manage risk include medical and lifestyle management, and cardiac defibrillator implantation [4–6].

Policy around search and return of SF will be informed by clinical actionability, including the positive predictive value of SF in populations not ascertained for the associated health condition. This question is more amenable to study in ICC than some other disorders, since disease expression can be assessed in any adult variant carrier through detailed clinical assessment at the time of presentation. In the context of manifest disease, family-based studies demonstrate the incomplete and variable penetrance of ICC variants [7–9]. While genotype-first approaches overcome the ascertainment bias inherent to case series and family-based studies, phenotypic features described are often derived from retrospective electronic medical records, lack specific ICC-targeted investigations, and hence may underestimate the magnitude of any effect [10–14]. The few reports of ICC SF disclosure highlight challenges with interpreting variants and correlating them with clinical findings and family history, and with patient management [15–17]. Pathogenicity assessment remains a challenge, especially in heterozygous disorders caused by missense variants [18].

The majority of patients, public and healthcare professionals support the return of ‘actionable’ SF, however, appreciation of the complexity of genomic information may temper attitudes to the scope of findings [19]. Increasingly, GS is expanding to ‘healthy’ cohorts; while recent initiatives have sought consent to return SF in variable gene lists, earlier ones did not, posing dilemmas relating to ethical recall-by-genotype studies [20].

Here we report on an approach to disclosure and targeted clinical evaluation of ICC SF in an established rare disease study cohort: a recall-by-genotype double blind, case-control feasibility study which aimed to assess the penetrance of ICC gene variants in an unselected population, and explore the psychosocial and behavioural sequelae of clinical assessment. The study, Secondary Cardiac Findings Evaluation (SCARFE), piloted a two-stage recruitment approach, designed to disclose SF information in an expert setting, and to enhance participant autonomy [21] in participants who were not offered SF options at initial recruitment.

## Materials and methods

### NIHR BioResource for rare disease (BRRD)

BRRD (https://bioresource.nihr.ac.uk/rare-diseases/rare-diseases/) enrolled affected participants and their relatives across several different rare disease areas including infection and immunity, neuroscience, rare cancers and cardiovascular disease (Research Ethics Committee (REC) reference 13/EE/0325). Consent to BRRD included recontact for additional ‘Stage 2’ studies. The SCARFE study was approved by London Fulham research ethics committee (REC reference 17/LO/1579).

### Genome sequencing

GS of DNA from blood samples from BRRD participants was undertaken as reported elsewhere [22].

### Genomic analysis and participant re-identification

Twenty ICC genes robustly associated with HCM (*ACTC1, GLA, LAMP2, MYL2, MYBPC3, MYL3, MYH7, TNNI3, TNNT2, TPM1*), DCM (*LMNA, VCL, MYH7, TNNI3, TNNT2, TPM1*), ARVC (*DSC2, DSG2, DSP, PKP2*) and long QT syndrome (*KCNH2, KCNQ1, SCN5A*) were evaluated (Table 1) in sequence data from BRRD participants not known to have an ICC. Previously reported variants classified pathogenic in the context of autosomal dominant ICC (Oxford Medical Genetics Laboratory, OMGL; Partners Laboratory of Molecular Medicine [23]; or ClinVar), and truncating variants in genes where this class of variant is considered pathogenic, were extracted. Variants assigned pathogenic or likely pathogenic status were confirmed by Sanger sequencing by the BRRD. The TTN gene was not included since at the time of analysis, understanding of variant-disease association was incomplete and variants not considered actionable.

**Table 1:** Variants identified in genome sequence data: candidate gene variants considered ‘highly likely’ or ‘likely’ pathogenic.

Disease associated	Gene	Reference sequence	c.	p.	No. occurrences	Variant classification
ARVC	*DSC2*	NM_024422.3	c.1123C > T	p.(Arg375*)	1	Pathogenic
ARVC	*DSC2*	NM_024422.3	*c.2533G* *>* *T (variant not confirmed)*	p.(Glu845*)	*1*	*Likely pathogenic*
ARVC	*DSC2*	NM_024422.3	c.379G > T	p.(Glu127*)	1	Likely pathogenic
ARVC	*DSC2*	NM_024422.3	c.939_942delAGAG	p.(Arg313fs)	1	Likely pathogenic
ARVC	*DSC2*	NM_024422.3	c.959delA	p.(Gln320fs)	1	Likely pathogenic
ARVC	*DSG2*	NM_001943.4	c.1880-2 A > G	p.?	1	Pathogenic
ARVC	*DSG2*	NM_001943.4	c.2894_2897delAGAG	p.(Glu965fs)	1	Likely pathogenic
ARVC	*DSG2*	NM_001943.4	c.355C > T	p.(Arg119*)	1	Likely pathogenic
ARVC	*DSG2*	NM_001943.4	c.375dupT	p.(Leu126fs)	2	Pathogenic
ARVC	*DSG2*	NM_001943.4	c.603_604delAT	p.(Leu203fs)	1	Likely pathogenic
ARVC	*PKP2*	NM_004572.3	c.1035-1G > C	p.?	1	Likely pathogenic
ARVC	*PKP2*	NM_004572.3	c.1237C > T	p.(Arg413*)	1	Pathogenic
ARVC	*PKP2*	NM_004572.3	c.2146-1G > C	p.?	1	Pathogenic
ARVC	*PKP2*	NM_004572.3	c.2194C > T	p.(Gln732*)	1	Pathogenic
ARVC	*PKP2*	NM_004572.3	c.2378_2379dupCC	p.(Ser794fs)	1	Pathogenic
ARVC	*PKP2*	NM_004572.3	c.2478delT	p.(Ser826fs)	1	Pathogenic
DCM	*LMNA*	NM_170707.3	c.949G > A	p.(Glu317Lys)	1	Likely pathogenic
HCM	*GLA*	NM_000169.2	c.644A > G	p.(Asn215Ser)	1	Pathogenic
HCM	*MYBPC3*	NM_000256.3	c.1504C > T	p.(Arg502Trp)	3	Pathogenic
HCM	*MYBPC3*	NM_000256.3	c.1624G> C	p.(Glu542Gln)	1	Pathogenic
HCM	*MYBPC3*	NM_000256.3	c.2373dupG	p.(Trp792fs)	1	Pathogenic
HCM	*MYBPC3*	NM_000256.3	c.26-2A > G	p.?	2	Likely pathogenic
HCM	*MYBPC3*	NM_000256.3	c.3192dupC	p.(Lys1065fs)	1	Pathogenic
HCM	*MYBPC3*	NM_000256.3	c.3592dupG	p.(Ala1198fs)	1	Pathogenic
HCM	*MYBPC3*	NM_000256.3	c.772G > A	p.(Glu258Lys)	2	Pathogenic
HCM	*MYBPC3*	NM_000256.3	c.994G > T	p.(Glu332*)	1	Pathogenic
HCM	*MYL3*	NM_000258.2	c.170C > G	p.(Ala57Gly)	2	Likely pathogenic*
HCM	*MYH7*	NM_000257.2	c.4066G > A	p.(Glu1356Lys)	1	Likely pathogenic
HCM	*TNNI3*	NM_000363.4	c.433C > T	p.(Arg145Trp)	2	Pathogenic
HCM	*TNNI3*	NM_000363.4	c.434G > A	p.(Arg145Gln)	1	Likely pathogenic
HCM	*TNNI3*	NM_000363.4	c.484C > T	p.(Arg162Trp)	1	Likely pathogenic*
HCM	*TNNI3*	NM_000363.4	c.485G > A	p.(Arg162Gln)	1	Likely pathogenic*
LQTS	*KCNH2*	NM_000238.3	c.1744C > T	p.(Arg582Cys)	1	Likely pathogenic
LQTS	*KCNQ1*	NM_000218.2	c.1066C > T	p.(Gln356*)	2	Pathogenic
LQTS	*KCNQ1*	NM_000218.2	c.1780C > T	p.(Arg594*)	1	Pathogenic
LQTS	*KCNQ1*	NM_000218.2	c.1781G > A	p.(Arg594Gln)	1	Likely pathogenic
LQTS	*SCN5A*	NM_001099404.1	c.6017dupC	p.(Ser2007fs)	1	Likely pathogenic
Total number of participants					45	

No. occurrences = number of times variant found in the BRRD cohort (*n* = 7203). Variants reviewed and classified January–March 2017.

Codes of all participants harbouring a likely/highly likely pathogenic variant, together with variant details, were made available to the BRRD, who re-identified participants and assessed for eligibility: age 18–80 years, not known to have a condition associated with the SF, or that might confound clinical test interpretation, and matched with non-carrier controls by age, gender and primary disease cohort. Inclusion of matched non-variant carrier controls allowed us to approach BRRD participants without disclosing variant carrier status, to perform and analyse targeted tests while blind to genotype and to study impacts of the double blind approach on all participants.

### Participant approach

Eligible BRRD participants and controls were initially sent an ‘opt-out’ letter [21] (Supplementary Material). Participants (cases and controls) who did not opt out within 4 weeks were sent an invitation letter to SCARFE requesting reply to the BRRD team, using a code for the specific ICC associated with the variant. Contact details of participants who responded positively were passed to the SCARFE study team. Case/control status was blind to participants and SCARFE study team.

### Study visit

Study rationale, possible outcomes, clinical and lifestyle consequences of significant clinical findings and presence of an ICC gene variant for the participant and relatives, were discussed in full with potential SCARFE participants before written informed consent was taken. A cardiac-focussed medical history and three-generation pedigree were recorded. Clinical evaluation was performed by a single experienced operator using the same equipment according to the ICC associated with the variant detected. Clinical tests were interpreted by a cardiologist specialised in ICC and a diagnostic opinion recorded, prior to unblinding of case/control status. All findings were disclosed to participants the same day, with letter to participant general practitioner and recommendations regarding clinical follow-up in a National Health Service ICC clinic, and family cascade testing as appropriate, supported by family cascade letters. Variants were re-confirmed in a fresh sample by OMGL prior to cascade testing.

### Qualitative data collection and analysis

Semi-structured interviews were undertaken by the first author between 6 and 12 months post visit using a guide developed through clinical experience and literature review. Interviews aimed to explore participant perceptions of being approached and of the study visit, their reactions to the information received, whom they had discussed the visit with, and reasons for any lifestyle changes. Interviews were audio recorded and verbatim transcripts analysed thematically [24] by the first two authors.

## Results

### Genetic analysis

Pathogenic/likely pathogenic variants within ICC genes were detected and confirmed in 44/7203 (0.61%) BRRD participants: numbers of participants carrying a variant associated with each disease: 16 ARVC, 1 DCM, 21 HCM, 6 LQTS (Table 1). Sanger sequencing failed to confirm one variant in one BRRD participant, who was not invited to SCARFE.

### Participants

**Fig. 1:**
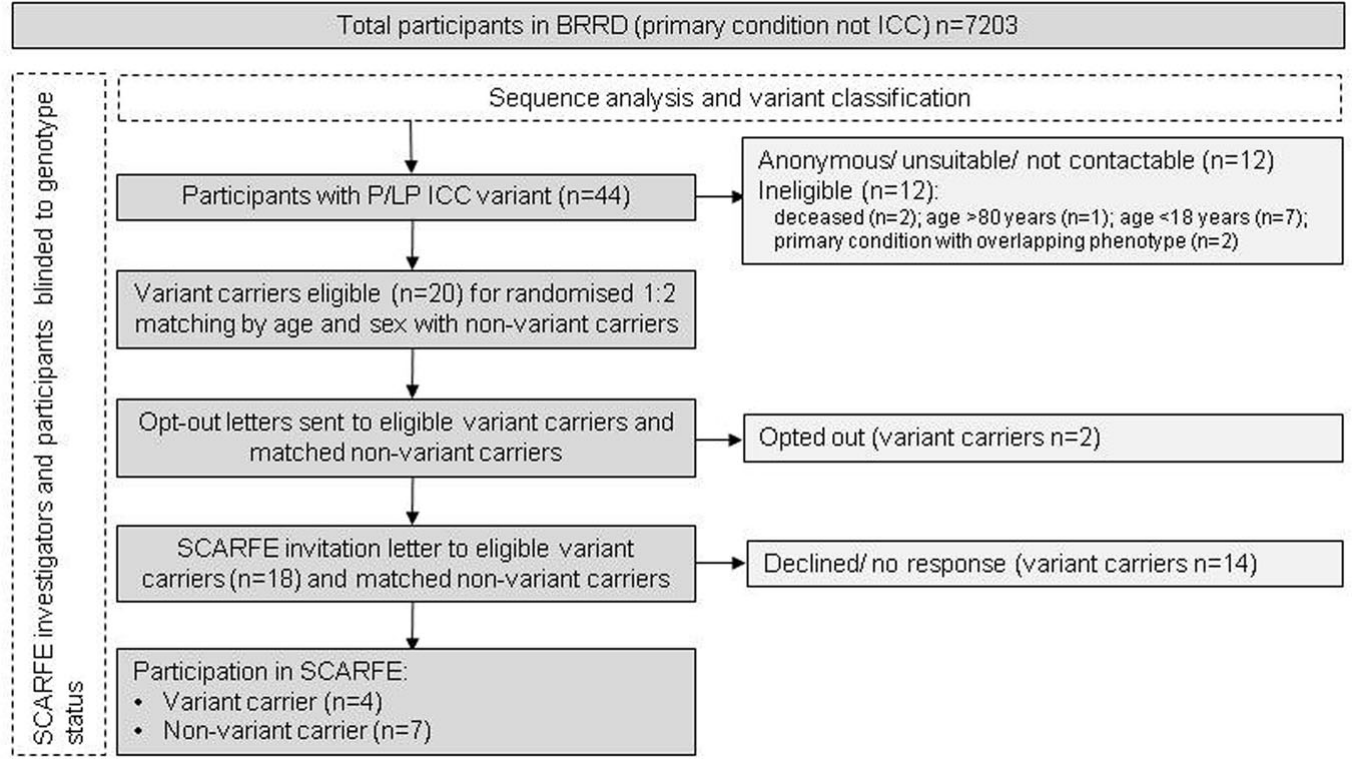
SCARFE recruitment by genotype process, showing the numbers of BRRD participants at each stage.

Twelve of the 44 variant carriers were ineligible and a further 12 could not be contacted or were considered unsuitable by the referring clinician. Initial opt-out letters were sent to 20 variant carriers, and returned by two. Invitation letters were sent to the remaining 18 eligible variant carriers, of whom 14 actively declined or did not respond to invitations. Four (mean age = 60) eligible variant carriers attended a study visit and participated. Approach and numbers at each stage are shown in Fig. 1. Variants carried by all four were in genes associated with HCM.

Seven non-carrier controls (mean age = 55) participated; four were matched to a case who declined or did not respond, and for one case, two controls were invited but declined or did not respond. Ten participants who attended a study visit took part in a semi-structured interview; one did not respond to requests.

### Clinical findings, family history and genotype

Genetic variants, demographic data and clinical findings of variant carriers who attended for assessment are shown in Table 2.

**Table 2:** Characteristics and clinical findings of variant carriers participating in SCARFE.

ID	Age (gender)	Echo MWT (cm)	Echo LA dimension (cm)	Echo comments	ECG comments	BP (mm Hg)	Prior family history HCM/SCD	Clinically affected status (blind to genotype)	Variant	ACMG classification [18] (reassessment after SCARFE participation)	Relatives at known risk after SCARFE participation (gene test)	Recommendations
B3	70 years old (F)	1.2	3.0	No hypertrophy, normal contractility. Slightly enlarged atria	Small voltages; not suggestive of HCM	138/70	No	Unaffected	c.2373dupG, p.(Trp792fs) *MYBPC3* NM_000256.3	Pathogenic	Parent (tested); sibling (tested); child (tested)	5-yearly follow-up; cascade
B13	46 years old (M)	1.5	3.5	Slightly hypertrophied mid-septum, suspicious for HCM. Unremarkable contractility	Sinus bradycardia 55 bpm; normal voltages with no criteria for LVH	113/65	Yes: sibling with HCM; no gene test	Affected	c.3592dupG p.(Ala1198fs) *MYBPC3* NM_000256.3	Pathogenic	Siblings *n* = 2 (testing awaited); children *n* = 2 (tested)	HCM risk stratification, annual follow-up, cascade
B20	66 years old (F)	1.2	3.0	Slight septal bulge, insufficient for diagnosis	Small Q waves in L3, otherwise normal	155/72	Yes: 2 FDRs died suddenly in 70s	Unaffected	c.170C > G p.(Ala57Gly) *MYL3* NM_000258.2	Likely pathogenic (VUS)	Child (testing awaited); second degree relatives with deceased intervening relative *n* = 3 (2 tested, 1 test awaited)	5-yearly follow-up; cascade
B17	60 years old (F)	1.1	3.4	Normal septal thickness and contractility	No voltage criteria for LVH	122/72	No	Unaffected	c.170C > G p(Ala57Gly)*MYL3* NM_000258.2	Likely pathogenic (VUS)	Siblings *n* = 3 (1 tested, 2 not yet sought testing); children *n* = 2 (1 tested, 1 not yet sought testing)	5-yearly follow-up; cascade

Variants carried by all were associated with HCM. All participants were of European ancestry.

*MWT* maximum wall thickness, *LA* left atrial, *bpm* beats per minute, *FDR* first-degree relative, *LVH* left ventricular hypertrophy, *BP* blood pressure.

B3 is a 70-year-old asymptomatic woman. Study visit echocardiogram showed septum measurement at the upper limit of normal with normal left ventricular size and systolic function, no left ventricular outflow tract obstruction, no systolic anterior motion, normal atria and no voltage criteria for left ventricular hypertrophy (LVH) on ECG. B3 has no known family history of HCM or sudden cardiac death. Clinical verdict was that B3 was not affected with HCM. After unblinding, B3 was found to carry a well-established pathogenic HCM variant in *MYBPC3* [25] and was referred for clinical care: clinical cardiac magnetic resonance (CMR) imaging confirmed absence of LVH. B3 has three living adult first-degree relatives who have subsequently been tested, of whom one has the variant and is clinically unaffected.

B13 is a 46-year-old asymptomatic man employed in a profession requiring medical certification. Genotype-blind echocardiogram showed mild hypertrophy in the mid-septum with normal left ventricular size and systolic function, no left ventricular outflow tract obstruction, no systolic anterior motion and normal atria; ECG showed no criteria for LVH. One sibling had a prior diagnosis of HCM; no clinical genetic testing had been performed. Following the sibling’s diagnosis, B13 was clinically screened in his 30s, considered unaffected and discharged. Clinical verdict was that B13 was affected with HCM. He was found to carry a novel variant in *MYBPC3* predicted to result in premature termination of translation resulting in haploinsufficiency, a known disease mechanism in HCM. Subsequent clinical CMR confirmed asymmetric septal hypertrophy (MWT 1.8 cm) with large areas of patchy fibrosis with late gadolinium enhancement. Twenty-four hours ECG and exercise test were normal. Family testing is ongoing.

B20 is a 66-year-old asymptomatic woman with normal left ventricular size and systolic function, no left ventricular outflow tract obstruction, no systolic anterior motion, normal atria and normal ECG. Two first-degree relatives suffered sudden deaths both at age 76. Clinical verdict was that B20 was not affected with HCM. She was found to have a variant in *MYL3* considered likely pathogenic for HCM at the time of SCARFE genomic analysis: this variant is reported as pathogenic on ClinVar due to segregation and functional work in vitro [26] and in vivo [27], but has a higher than expected allele frequency (0.0072%) in gnomAD (r.2.1.1). After the study visit we reviewed the variant and considered evidence for pathogenicity uncertain, with further cardiac investigations not warranted. Cause of death in relatives was certified as cardiac failure and hypertension (parent); acute myocardial infarction, coronary atherosclerosis and myocardial hypertrophy and hypercholesterolaemia (sibling). Cascade testing is ongoing.

B17 is a 61-year-old asymptomatic woman. She has normal left ventricular size with normal wall dimensions and function, no left ventricular outflow tract obstruction, no systolic anterior motion and normal atria, normal ECG. She has no family history of HCM or sudden death. Clinical verdict was that B17 was not affected with HCM. B17 carries the same *MYL3* variant detected in B20. Cascade testing is ongoing.

Clinical data for the non-variant carriers are shown in Table 3. None was considered to have clinical features diagnostic of the ICC for which they had been enrolled as controls.

**Table 3:** Characteristics and clinical findings of control participants in SCARFE.

ID	Age (gender)	Matched case	Phenotype associated with variant carried by matched case	Echo MWT (cm)	Echo LA dimension (cm)	Echo comments	ECG comments	Blood pressure (mm Hg)	Clinically affected status
B5	48 years old (F)	Declined	HCM	1.0	3.3	Normal contractility, normal cavity size and wall thickness. Borderline increase in LA volume	ST-T wave changes in V2/V3 of uncertain significance; no voltage criteria for LVH	122/79	Unaffected
B2	42 years old (F)	Declined	DCM	0.8	3.7	Normal EF; normal cavity dimension	Normal	101/76	Unaffected
B16	46 years old (M)	B13	HCM	1.2	4.2	MWT upper limit of normal; large vessels in keeping with athletic training, normal diastolic function	T wave inversion in L3, AVF; slight QRS widening (120 ms); prominent U wave, PR interval short (88 ms). No obvious delta wave	146/98	Unaffected
B22	67 years old (F)	B20	HCM	1.2	3.6	Borderline localised proximal hypertrophy, slightly dilated aortic root in keeping with hypertension	No evidence of prior infarct. PR interval upper limit of normal; shallow T wave inversion in V2. No voltage criteria for LVH	134/77	Unaffected
B9	39 years old (F)	No response	HCM	0.8	2.5	Normal	Normal	130/75	Unaffected
B3M	63 years old (F)	Declined	LQTS	1.2	3.7	Normal	Normal ST segment and T wave. QTc 402 ms	132/80	Unaffected
B7	70 years old (F)	B3	HCM	1.2	3.8	MWT upper limit of normal; normal contractility	Normal; no voltage criteria for LVH	154/84	Unaffected

All participants were of European ancestry.

*MWT* maximum wall thickness, *LA* left atrial, *bpm* beats per minute, *LVH* left ventricular hypertrophy, *EF* ejection fraction, *QTc* QT interval (corrected using Bazett formula).

### Qualitative data analysis

#### Understanding of genetic health risks

Participants talked at length about the primary health condition (for which participants were enrolled in the BRRD) in them/their child, and the multiple psychosocial and practical burdens it had imposed on the family. No participant had yet received a genetic diagnosis. Experience with the primary health condition had informed participants about concepts such as inherited disease and risk, variable penetrance and expressivity, and diseases for which treatment is available but no cure. Some participants considered that leading a healthy lifestyle could mitigate disease development in general, despite presumed genetic risk. One of the participants carrying a variant subsequently reclassified already had experience of a variant of unknown significance (VUS) found in relation to the primary condition; she explained her understanding of the meaning of uncertainty in genetic disease:

‘This unknown significant stuff, I’d had a lot of time to get my head around with [child] having something, which they had downplayed considerably. That’s quite different from this unknown variation. I thought it was a very binary answer. It was either, ‘Yes,’ or, ‘No,’ and because I knew that I hadn’t got any heart problems, because my ECG had been fine and everything else, then, I just assumed that I was a no. It never occurred to me’. (B17).

#### Motivation for and satisfaction with participation in SCARFE

Participants agreed to take part for altruistic reasons as well as personal and family reasons. Only two (of ten) participants recollected the two-stage SCARFE approach. Of interest given the low response rate, one participant had assumed that she was ‘one of many’, and that her participation was not critical. Although some participants reported anxiety about the tests and what they might show, all were satisfied with the study processes before, during and after the study visit; they valued the ‘personalised approach’, ‘empathy’ and ‘clarity’, at the study visit, and availability of the study team post visit. All participants had clear and accurate recollection of personal genetic and cardiac findings from their SCARFE visit.

#### Risk perception

With one exception, participants did not consider, prior to attending the study visit, that they would have an ICC. This low perception of risk was informed variously by lack of symptoms, fitness levels or regular well-person checks in primary care with no heart problems noted. One participant perceived his risk as high: he appreciated that he could be genetically affected but clinically unaffected (earlier tests showed no signs of HCM). Another participant stated in hindsight (during interview) that she had expected to ‘have it’ and this was in some way entwined in her thoughts about her deceased child, albeit whose condition was unrelated to ICC:

‘I thought I had it…I didn’t think, oh my God I’m going to die or anything like that. I just thought, oh this is linked, perhaps, to [child]. I know [child] was so complex, so it was just, oh I thought, oh well perhaps we can help that area as well’. (B3)

However, her very active lifestyle, wellness and parents’ longevity were, for her, in contradiction with the possibility that she might have an ICC.

#### Personal consequences of participation

No participant regretted participation in SCARFE. For the one participant who found to have clinical signs of ICC, the finding provoked anxiety about the risk posed to continuing his occupation—a source of satisfaction, financial security and fulfilment of ambition since childhood—and period of uncertainty, while he underwent further assessment mandated by his employer. Having cleared this process by the time of interview, his concerns were then about the risk of HCM to his children, already burdened by significant illness. The other case participant with a pathogenic variant (clinically unaffected) found it hard to conceptualise her risk:

‘Am I lucky to be here? How come something didn’t happen?..it’s made me wary that I mustn’t go over the limit..so yes, it’s quite difficult that side’. (B3)

In two control participants, there were unexpected clinical findings unrelated to the ICC. One participant was found to have a subtly abnormal ECG, which the study team reported to his primary care doctor; he described subsequent potential cardiac symptoms on exercise and further medical investigation and monitoring. A further control participant believed she had suffered a ‘heart attack’ decades earlier. At her study visit, she was reassured that her heart showed no evidence of damage, and this evoked several reactions:

‘Then to find out that all those years when I couldn’t do things there was nothing wrong with my heart. A bit angry as well, because it did affect my life a lot… I go from being relieved—well, I am relieved, I’m very pleased about my heart’. (B22)

Other participants also recalled relief at learning their hearts were healthy, but only one (a case) described making positive lifestyle changes as a result of the study, wishing to maintain her health. One participant had ‘good intentions’, others felt they already had a healthy lifestyle.

#### Family communication and outcomes

The four case participants were advised to inform immediate relatives and were provided with cascade letters. While all four had informed relatives, they had not always found it straightforward depending on individual relatives’ personalities, circumstances and family dynamics. One participant with an elderly parent described her reasons for not informing them; another had arranged genetic testing with the parent’s care home. One participant knew of aunts and uncles who, with their descendants, could be at risk, but had no contact with them.

#### Responses of heath care system

Aside from SCARFE, participants in general reported personal experience of strains on primary care practice. Relatives were distributed around the country. In two families, at-risk relatives had accessed appropriate testing/screening if they had sought it, but in two, relatives had struggled to persuade primary care physicians to refer for evaluation:

‘[Relative’s primary care doctor] was not helpful at all, no. She rang them up and sorted that out for herself. [Another relative’s doctor] wouldn’t refer him. He said he would do a blood test for him, but if he found that he’d got anything, he still wouldn’t refer him’. (B20)

Other relatives were told they would have to wait many months for an appointment. One relative had been able to access cardiac screening but not genetic testing. Primary care doctors were perceived as having limited understanding of genetic risk; this, and delays to appointments, had caused frustration and sometimes anxiety for participants and, reportedly, for relatives.

## Discussion

To our knowledge, this is the first study reporting on a systematic approach to disclosure of SF ICC, with targeted but genotype-blinded clinical evaluation and detailed exploration of sequelae of disclosure. Using this approach we have been able to explore many elements of a policy to look for and report ICC SF, critical for informing policy around SF. The possibility that SF might cause high levels of anxiety has been a concern among healthcare professionals and publics [19]. Distress or anxiety following SF disclosure should be factored into the risk/benefit equation, particularly if SF prove to have relatively low penetrance in unselected populations. Several studies [28–31], including one involving LQTS findings [32], have shown that this concern may be unfounded; but case reports show how anxiety may lead to disengagement with healthcare services [16]. Failure to assimilate SF information for whatever reason might also impede realisation of clinical utility: ‘patients’ (SF recipients) need to make cognitive and emotional adjustments to health risk information to enable relevant decisions about their own healthcare and lifestyle, and communicate risk information successfully to relatives [33]. Participants in our study denied ongoing anxiety caused by the SF per se, and were able to act as recommended, attending for further clinical testing and informing relatives. However, we cannot discount the possibility that among those who declined participation were some who might have found adaptation to new risk information challenging. Participants also demonstrated clear understanding of genetic health risks and concepts such as variable penetrance, informed by their experiences with rare disease; they valued information provided as part of the study design and the availability of continued access to the study team after the study visit.

Maximising the clinical utility of SF requires that healthcare services are able to enact appropriate clinical follow-up and cascade testing [34]. Unless carriers and their relatives are able to access genetic testing, clinical evaluation and follow-up, a search and disclosure policy for SF is of limited utility. In the present study, we found variable primary care responses to relatives’ request for a referral even when supported by a clinical cascade letter: long waiting times and for one participant, a cardiology assessment offered before a genetic appointment. While variability is also seen in ICC clinical practice in the UK, healthcare professional engagement challenges may be exacerbated in families in which no one is clinically affected by the condition associated with the SF. Greater education and resourcing of primary care physicians may be required for this aspect of preventative medicine.

In this study, risk perception was low for all participants who had no family history of ICC. Risk perception was informed by lack of symptoms and high current or former levels of activity, as we have observed in an earlier study of pre-symptomatic testing in ICC [35]. These factors, as well as general health checks in primary care mentioned by some, do not preclude clinical signs of ICC. Further research is needed to understand interactions between risk perception and outcomes of SF disclosure.

The study identified one variant carrier with HCM, who had been previously clinically assessed after a diagnosis of HCM in a first-degree relative, and discharged. Had genetic testing been used in the proband and in family evaluation, it is likely the variant would have been detected and the participant identified as a carrier warranting periodic reassessment. This example highlights the value of clinical genetic testing and cascading for identifying ICC risk in relatives, for their personal health and risk assessment of employees in regulated professions. Incomplete utilisation of clinical genetic testing in cardiomyopathy (DCM) was also found in a genetics-first EMR-based study [36]. Genetic testing is a Class 1 recommendation for most ICC in a proband with clinical suspicion of disease [37]. However, where genetic testing is either not available or not informative, a single clinical evaluation of relatives in young/mid adulthood is insufficient to rule out risk of ICC.

A second carrier of a pathogenic (HCM) variant was clinically unaffected; cascade testing has clarified that her surviving parent and that parent’s many descendants are at population risk. The variant in question, a frameshift in *MYBPC3*, is considered unambiguously pathogenic, occurring at high frequency in the Dutch HCM population [25] and OMGL. Clearly it is possible that SF disclosure will fail to find evidence of disease in a family, although we cannot establish whether the deceased parent’s relatives have or are at risk of HCM, due to loss of contact with their descendants.

The study highlights challenges with variant assessment: the *MYL3* variant (c.170C > G p.Ala57Gly) detected in our study was considered a pathogenic SF by Natajaran et al. [12] prior to reclassification. Variant interpretation remains inexact and subject to change, presenting a particular challenge in the context of SF when the pretest probability of disease is low [38]; expert laboratory input and genetic counselling will be critical to ensure patient understanding and appropriate clinical follow-up. Conversely, a variant considered a VUS at analysis but subsequently reclassified as pathogenic would not be disclosed.

A dilemma with recall-by-genotype studies is how to approach variant carriers to participate without divulging significant but unexpected risk information or compromising their right not-to-know [20]. This is a concern for studies in which participants had not originally consented to return of SF. Our study design used two strategies to recall participants: a two-step approach as proposed by Beskow et al. [21], whereby an initial letter asked participants in the original study to opt out if they did not wish to learn health or genetic information additional to what they already knew about. Two BRRD participants opted out at this stage, suggesting that this approach promoted autonomy for some, however, most who accepted participation were unable to recall this letter as distinct from the study invitation letter. Second, we used a case/control strategy, in which participants could learn more about the study and possible implications with specialist genetic counselling, before deciding whether to proceed with clinical screening or learning their genetic status. While this strategy achieved its aim, it may have contributed to low recruitment rate. Further research is necessary to understand optimal approach in recall-by-genotype studies.

### Study limitations

The main limitation is the small number of eligible participants and low response rate. From a cohort of over 7000 participants, we are unable to draw conclusions about ICC penetrance in the general population; given the rarity of pathogenic variants in these genes, meaningful estimates of ICC SF penetrance will require a very large sequenced population together with detailed cardiac phenotyping. While we have little information about individual reasons for declining, from participant interviews we can infer factors that may have contributed to low participation rate: study participants were recruited from a study investigating rare disease; many have experienced a high burden of disease, requiring high levels of care and multiple hospital appointments. This may have influenced participation rate, psychosocial and behavioural responses compared with a ‘healthy’ population. In addition, the case-control approach, while chosen for optimal design as well as to protect participants, restricted promotion of the possible health benefits of participation. In this study, many people chose not to pursue SF, however, interest in taking part in a research study may not be comparable with uptake of SF in other contexts.

In conclusion, we have piloted a protocol for return and clinical evaluation of putatively pathogenic ICC variants as SF to participants in a study where participants had not been asked to consent for SF. We show that ICC SF can be returned and acted upon without undue anxiety in recipients, but that UK clinical healthcare services are not fully utilising genetic testing in the context of ICC diagnosis, and may need additional resourcing and education to enact family cascading following SF disclosure. The finding of an unambiguously pathogenic ICC SF in a healthy participant highlights that ICC SF cannot be considered deterministic.

## Supplementary information

BioResourceStage2pre-opt-out.pdf
