# Supplementary material for: Secondary findings in inherited heart conditions: a genotype-first feasibility study to assess phenotype, behavioural and psychosocial outcomes
Source: Eur J Hum Genet. 2020 Jul 20;28(11):1486–96. doi: 10.1038/s41431-020-0694-9 (PMC7576165; doi:10.1038/s41431-020-0694-9)
Supplement: Supplementary file 1 — BioResourceStage2pre-opt-out.pdf [file 41431_2020_694_MOESM1_ESM.pdf]

NIHR BioResource
Rare Diseases

NHS
National Institute for Health Research

Participant name NHS Number [if available]

Address

Date

Dear [name of participant],

Title of the Research Study: NIHR BioResource – Rare Diseases Study

Thank you for your continued membership in the NIHR BioResource – Rare Diseases.

You may recall that when you consented to join the NIHR BioResource – Rare Diseases, you agreed to be invited to take part in future medical research studies.

It is possible that some of these studies might give you **information about your current or future health, or genetic information which can be passed from parent to child**. **This information may be unrelated to the known rare disease in you/your** family.

If you **DO NOT** wish to be contacted about studies which might provide you with health or genetic information unrelated to your/your family's condition, please complete and return the reply slip attached. We will record on our database that you have declined to receive such invitations and you will not receive any further information about such studies.

If you **DO** wish to receive invitations to studies where you might receive additional health or genetic information, you do not have to do anything. If we have not heard from you by [insert date 4 weeks after postal date] we will assume that you are happy to receive such invitations.

It is entirely up to you whether you take part in any of the studies we might invite you to. Deciding not to take part will have no effect on the healthcare that you receive or your continued participation in the NIHR BioResource – Rare Diseases. All invitations you receive will include a detailed Information Sheet which will explain what the study involves. Where a study might provide you with additional health or genetic information, procedures will be in place to provide you with any advice and support you may require.

If you would like any further information, or you would like to discuss this letter in more detail please contact us on Freephone 0800 0853650 or by email: rarediseases@bioresource.nihr.ac.uk. You can change your mind about receiving these invitations at any point; please contact us using the contact details above to update your preference. Thank you for taking the time to read this letter.

Yours sincerely

Professor Willem H Ouwehand MD PhD FRCPath FMedSci

Director NIHR BioResource – Rare Diseases

Additional Health and Genetic Information Opt-Out

Complete this form only if you DO NOT want to receive invitations to studies where your health or genetic information may available to you.

Please return it in the pre-paid envelope to NIHR BioResource – Rare Diseases study by xx-xx-xxxx, otherwise it will be assumed that you are willing to receive invites to studies where you may receive feedback about this information.

I do not want to be contacted by the BioResource Team about any study that might provide **information about my current or future health, or genetic information which can be passed from parent to child**, that is not related to the known health condition in me/my family [*delete as appropriate*].

Your signature ………………………………………………………………….…….

Your name (Please print) …………………………………………………………………….

Your date of birth …./…./….

Date …./…./….
